# Regionale Unterschiede in der Entfernung zur fachärztlichen rheumatologischen Versorgung in Deutschland

**DOI:** 10.1007/s00393-025-01641-z

**Published:** 2025-03-25

**Authors:** Carlo Veltri, Nora Baer, Martin Aringer, Thorsten Eidner, Jörg Henes, Katja Thiele, Johanna Callhoff, Katinka Albrecht

**Affiliations:** 1 Programmbereich Epidemiologie und Versorgungsforschung, Deutsches Rheuma-Forschungszentrum (DRFZ) Berlin, Charitéplatz 1, 10117 Berlin, Deutschland; 2 Rheumatologie, Medizinische Klinik und Poliklinik III, Universitätsklinikum und Medizinische Fakultät Carl Gustav Carus, Technische Universität Dresden, Dresden, Deutschland; 3 Klinik für Innere Medizin III – Rheumatologie/Osteologie, Universitätsklinikum Jena, Jena, Deutschland; 4 Zentrum für Interdisziplinäre Rheumatologie, klinische Immunologie und Autoimmunerkrankungen (INDIRA), Department für Innere Medizin II, Universitätsklinik Tübingen, Tübingen, Deutschland

**Keywords:** Regionale Versorgung, Versorgungsforschung, Rheumatologie, Rheumatische Erkrankungen, Patientenmobilität, Regional care, Health services research, Rheumatology, Rheumatic diseases, Patient mobility

## Abstract

**Hintergrund:**

Eine rheumatologische Versorgung sollte für Menschen mit entzündlich rheumatischen Erkrankungen in allen Regionen Deutschlands verfügbar sein. In dieser Arbeit wird die Entfernung zur rheumatologischen fachärztlichen Versorgung kartographisch dargestellt.

**Methodik:**

Fachärzt:innen für Rheumatologie, die zum 31.12.2023 im Bundesarztregister verzeichnet waren, wurden Postleitzahlregionen zugeordnet. Die mittlere Luftlinienentfernungen von einer Postleitzahlregion zur nächstgelegenen Region mit einer rheumatologischen Praxis wurde berechnet. Von den an der Kerndokumentation teilnehmenden Rheumazentren wurde die mittlere Entfernung vom Wohnort für die betreuten Patient:innen berechnet.

**Ergebnisse:**

Es sind 25 % aller Postleitzahlregionen > 20 km Luftlinie von einem PLZ-Kreis mit rheumatologischer Praxis entfernt. Vor allem in Mecklenburg-Vorpommern, Niedersachsen und Schleswig-Holstein gibt es größere Gebiete mit geringer Versorgungsdichte. In der Kerndokumentation ist der Wohnort im Median zwischen 10 km (Berlin) und 40 km (Tübingen) von der rheumatologischen Einrichtung entfernt.

**Schlussfolgerung:**

In ländlicheren Regionen und für eine spezialfachärztliche Versorgung müssen lange Anfahrtswege zurückgelegt werden, um eine fachärztliche rheumatologische Versorgung zu erreichen.

## Abstract (en)

**Background:**

Rheumatological care should be available for people with inflammatory rheumatic diseases in all regions of Germany. In this article the distance to rheumatological specialist care is mapped.

**Methodology:**

Specialists in rheumatology listed in the German Federal Medical Register as of 31 December 2023 were assigned to postal code regions. The mean linear distance from a postal code region to the nearest region with a rheumatology practice was calculated. The mean distance from the places of residence of patients was calculated for the rheumatology centers participating in the German national database.

**Results:**

Of the postal code regions 25% are > 20 km linear distance away from a postal code district with a rheumatological practice. Especially in Mecklenburg-Western Pomerania, Lower Saxony and Schleswig-Holstein there are larger areas with a low density of care. In the German national database, the median distance between the patient’s place of residence and the rheumatological facility is between 10 km (Berlin) and 40 km (Tübingen).

**Conclusion:**

In rural areas and for specialized rheumatology centers patients must travel long distances to reach a specialized rheumatological care.

Gerade in der Rheumatologie stellen Anfahrtswege zur nächsten Praxis aufgrund eingeschränkter Mobilität einen wichtigen Aspekt des Patient:innenwohls und der allgemeinen Versorgungslage dar. Die Fahrtwege variieren jedoch regional stark, sodass sich Versorgungsdefizite v. a. in ländlichen Regionen ergeben. In diesem Artikel beleuchten wir diese regionalen Unterschiede.

## Einleitung

Regionale Versorgungsunterschiede werden in der Rheumatologie häufig benannt, wenn Versorgungsengpässe diskutiert werden. Im neuen Memorandum der Deutschen Gesellschaft für Rheumatologie und Klinische Immunologie e. V. (DGRh) wird auf eine geringe Versorgungsdichte an Fachärzt:innen (FÄ) für Rheumatologie in strukturschwächeren Regionen wie dem Saarland und Thüringen hingewiesen [1].

Die bisherigen Auswertungen basieren auf der Anzahl an FÄ für Rheumatologie pro 100.000 Einwohner bzw. Erwachsene auf Bundeslandebene [1, 2]. Gerade in großflächigen Ländern wie Niedersachsen und Brandenburg werden kleinteiligere regionale Unterschiede nicht abgebildet. Es gibt wenig Daten dazu, welche Anfahrtswege Patient:innen mit entzündlich rheumatischen Erkrankungen auf sich nehmen müssen, um die fachärztliche rheumatologische Versorgung zu erreichen. Aus der bundesweiten Kerndokumentation der regionalen kooperativen Rheumazentren ist bekannt, dass jede zweite Patient:in einen Anfahrtsweg von mehr als 20 km hat, deutlich länger für Land- im Vergleich zu Stadtbewohner:innen [3].

In dieser Arbeit wird die Entfernung zu vertragsärztlich tätigen FÄ für Rheumatologie auf 5‑stelliger Postleitzahlebene abgebildet. Von den rheumatologischen Einrichtungen aus der bundesweiten Kerndokumentation werden die mittleren Entfernungen vom Wohnort der Patient:innen für die jeweiligen rheumatologischen Praxen und Klinikambulanzen dargestellt.

## Methodik

Von der Kassenärztlichen Bundesvereinigung (KBV) wurden alle vertragsärztlich tätigen Fachärzt:innen (FÄ) für Innere Medizin und Rheumatologie, die zum 31.12.2023 im Bundesarztregister verzeichnet waren, anhand der Postleitzahl der rheumatologischen Praxis den Postleitzahlregionen (5-stellig) in Deutschland zugeordnet. Die Daten wurden uns ohne personenbezogene Angaben als 1/0-kodierte Tabelle (FÄ in Postleitzahlregion vorhanden/nicht vorhanden) zur Verfügung gestellt. Durch einen von Open Street Map [4] bereitgestellten Datensatz wurden die Postleitzahlen durch ihren jeweiligen geografischen Mittelpunkt beschrieben. Anschließend wurde für jede Postleitzahl, in welcher sich keine vertragsärztliche FÄ für Rheumatologie befindet, mithilfe der Statistik-Software R [5] die minimale Luftliniendistanz zur nächstliegenden Postleitzahl mit einer Praxis berechnet, um so eine Annäherung für die relativen Unterschiede in den regionalen Entfernungen zur nächstliegenden Praxis zu erhalten.

In der bundesweiten Kerndokumentation der regionalen kooperativen Rheumazentren werden jährlich versorgungsrelevante Daten von über 13.000 Patient:innen mit entzündlich rheumatischen Erkrankungen, die fachärztlich rheumatologisch betreut werden, erfasst. Die Patient:innen werden neben vielen anderen Angaben gefragt: „Wie weit ist es von Ihnen zu Hause bis zu dieser rheumatologischen Praxis/Klinik? Antwortmöglichkeit: ca. xx Kilometer.“

Von den 13 rheumatologischen Einrichtungen (3 Einzelpraxen, 3 Gemeinschaftspraxen, 1 Versorgungskrankenhaus und 6 Universitätskliniken), die im Jahr 2022 an der Kerndokumentation teilnahmen, wird für die in die Kerndokumentation eingeschlossenen Patient:innen die Entfernung vom Wohnort als Mittelwert, Median, Interquartilsabstand und 90 %-Quantil in km berichtet. Median und 90 %-Quantile sind in einer Deutschlandkarte abgebildet. Personen mit fehlenden Angaben zur Entfernung wurden von der Analyse ausgeschlossen.

## Ergebnisse

### Regionale Entfernungen zur nächstgelegenen FÄ für Rheumatologie in Deutschland

**Abb. 1:**
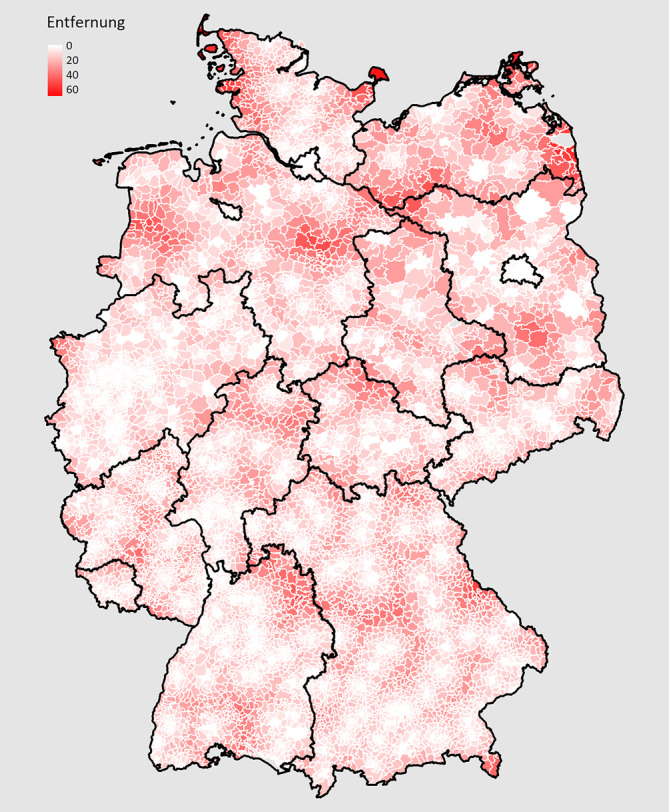
Entfernung zum nächstgelegenen PLZ-Kreis mit mindestens einer vertragsärztlich tätigen FÄ für Rheumatologie (Luftliniendistanz in Metern). Dargestellt ist die Luftliniendistanz in Metern der PLZ-Bereiche (ihrer geografischen Mittelpunkte) zum nächsten PLZ-Bereich mit rheumatologischer Praxis. Eine Distanz von 0 m (*weißer Bereich*) bedeutet, dass sich in diesem PLZ-Bereich eine rheumatologische Praxis befindet

Regionale Unterschiede in der Entfernung zur nächstgelegenen vertragsärztlich tätigen FÄ für Rheumatologie in Deutschland sind in Abb. 1 dargestellt; 25 % aller Postleitzahlregionen sind mehr als 20 km von einem PLZ-Kreis mit rheumatologischer Praxis entfernt. Regionen mit Entfernungen über 40 km liegen v. a. in den nördlichen Bundesländern, in Mecklenburg-Vorpommern, in Niedersachsen und in Schleswig-Holstein, aber auch in Brandenburg, Sachsen und auf kleinere Regionen begrenzt auch in anderen Bundesländern.

## Entfernung für Patient:innen zu den Rheumazentren der Kerndokumentation

**Abb. 2:**
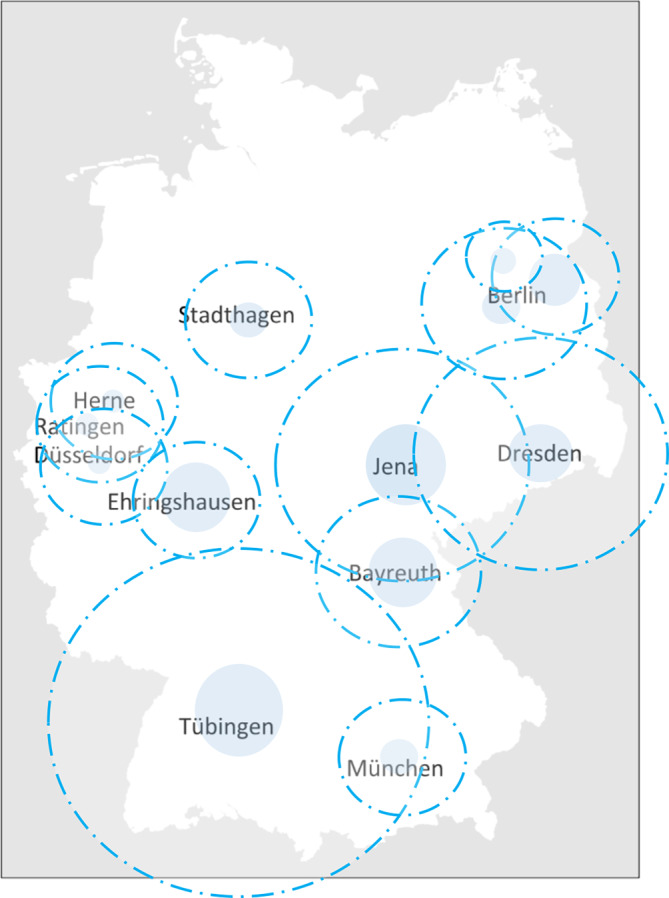
Entfernung für Patient:innen zu den Rheumazentren der Kerndokumentation. Der Radius der *blauen Kreise* entspricht dem Median der Entfernung zur jeweiligen Einrichtung, der Radius der *Kreise mit gestrichelten Linien* sind die 90 %-Quantile – d. h. z. B. für Ratingen: 50 % der Patient:innen wohnen 20 km oder weiter entfernt von der Praxis, und 10 % wohnen weiter als 45 km entfernt

Für die teilnehmenden Patient:innen aus der Kerndokumentation beträgt die mittlere Entfernung zu ihrer rheumatologischen Praxis oder Klinikambulanz 22 km bis 68 km (Tab. 1). In den Großstädten Berlin und München haben die Patient:innen den kürzesten Anfahrtsweg. Weite Wege legen Patient:innen zurück, die in Bayreuth, Jena und Tübingen betreut werden. Einige Einrichtungen haben Spezialambulanzen, die von weiter entfernt wohnenden Patient:innen mit seltenen Erkrankungen aufgesucht werden. Dies trifft z. B. für Tübingen (Behçet Ambulanz) zu. Hier wohnen 10 % der Patient:innen weiter als 150 km vom Rheumazentrum entfernt (Abb. 2).

**Tab. 1:** Entfernung vom Wohnort der Patient:innen zu den rheumatologischen Zentren der Kerndokumentation

Ort der rheumatologischen Einrichtung	Versorgungsstruktur	Anzahl Patient:innen^a^ (fehlende Angabe)	Mittelwert	Median	Interquartilsabstand (mittlere 50 %)	90 %-Quantil
Berlin-Steglitz	Gemeinschaftspraxis	232 (63)	22	10	14	30
Berlin-Charité	Uniklinik-Ambulanz	449 (111)	30	15	17	52
Stadthagen	Einzelpraxis	868 (36)	24	15	23	50
München	Einzelpraxis	749 (17)	28	15	15	50
Düsseldorf	Uniklinik-Ambulanz	1967 (422)	29	17	28	60
Herne	Uniklinik-Ambulanz	3235 (24)	27	18	23	55
Ratingen	Gemeinschaftspraxis	2867 (16)	24	20	20	45
Berlin-Buch	Klinikambulanz	584 (87)	34	21	27	70
Dresden	Uniklinik-Ambulanz	293 (21)	41	25	50	100
Ehringshausen	Gemeinschaftspraxis	165 (12)	29	28	21	49
Bayreuth	Einzelpraxis	469 (9)	33	30	40	65
Jena	Uniklinik-Ambulanz	751 (8)	44	35	45	99
Tübingen	Uniklinik-Ambulanz	1258 (63)	68	40	55	150

^a^ Mit Angabe der Entfernung zur betreuenden Rheuma-Praxis/Klinik

## Schlussfolgerung

Die Darstellung der Entfernung zu den nächstgelegenen FÄ für Rheumatologie auf Postleitzahlebene veranschaulicht regionale Unterschiede in der fachärztlichen Versorgungsdichte in Deutschland. In der Hälfte der Regionen beträgt die (mittlere Luftlinien‑)Entfernung zwar weniger als 12 km, bei einem Viertel aller PLZ-Gebiete jedoch mehr als 20 km und bei 1 % mehr als 40 km. Die Daten zur Entfernung sind eine wertvolle Ergänzung zu den bisherigen Analysen zur fachärztlichen Versorgungsdichte pro Einwohner [1, 2]. Im internationalen Vergleich zeigt eine Studie zu Fahrzeit und Reisekosten von Patient:innen mit rheumatoider Arthritis in Dänemark durchschnittliche Gesamtfahrzeiten (Hin- und Rückweg) von etwas mehr als 1 h (im Mittel 73 min mit großer Variation) zur ambulanten rheumatologischen Einrichtung. Keinen signifikanten Unterschied gab es zwischen Stadt- und Landbewohnern. Bei Nutzung öffentlicher Verkehrsmittel war die Fahrtzeit etwas höher (im Mittel 81 min) als bei Nutzung des privaten Autos (69 min) [6]. Die Studie untersuchte im Gegensatz zu unseren Daten Fahrtzeiten und nicht Distanzen, diese deuten aber auf kürzere Anfahrtswege im Vergleich zu Deutschland hin. Weite Anfahrtswege erschweren die Erreichbarkeit der fachärztlichen Versorgung. Das Rheuma-VOR-Projekt in Niedersachsen hat gezeigt, dass gerade für ältere und immobile Patient:innen der lange Anfahrtsweg in die fachärztliche Praxis der häufigste Beweggrund war, einen Termin abzusagen [7]. Da in den nächsten Jahren keine Zunahme an fachärztlichen Kapazitäten zu erwarten ist [1], sind für Regionen ohne rheumatologische FÄ Versorgungsmodelle mit hybriden bzw. mobilen Sprechstunden ebenso wie Remote-Monitoring-Konzepte Optionen, um dennoch eine fachärztliche Versorgung zu ermöglichen. Das Rheumabus Screening-Projekt, welches in verschiedenen Städten durchgeführt wurde, wurde von der Bevölkerung sehr positiv angenommen, basiert aber bislang lediglich auf einem Modellprojekt [7] und kann nur bei entsprechendem Aufwand und Finanzierung erfolgen.

Die konkreten Anfahrtswege von Patient:innen aus der Kerndokumentation ergänzen diese Daten um patientenberichtete Distanzen, die von den Betroffenen zurückgelegt werden, um die teils sehr spezialisierte rheumatologische Versorgung der Rheumazentren in Anspruch nehmen zu können. Auch hier gibt es große Unterschiede in den Entfernungen, wobei die Metropolen deutlich kürzere Anfahrtswege ermöglichen.

## Limitationen

Viele Praxen nehmen derzeit aus Kapazitätsgründen keine Neupatient:innen an. Daher ist die Entfernung zur nächstgelegenen Einrichtung nicht das alleinige Kriterium, die fachärztliche Versorgung auch in Anspruch nehmen zu können. Patient:innen ohne fachärztliche Versorgung werden mit der Kerndokumentation nicht abgebildet. Es kann daher anhand dieser Daten nicht abgeschätzt werden, wie viele Menschen mit entzündlich rheumatischen Erkrankungen die fachärztliche rheumatologische Versorgung nicht erreichen. Aufgrund der teils spezialisierten Kliniken sind die zurückgelegten Distanzen aus der Kerndokumentation nicht repräsentativ für alle Patient:innen mit entzündlich rheumatischen Erkrankungen, sie geben jedoch Aufschluss über tatsächlich zurückgelegte Distanzen. Da von den Patient:innen nur die Distanz zur betreuenden Einrichtung, aber nicht der Wohnort angegeben wird, können wir keine Aussage treffen, ob zwischen dem Wohnort und der betreuenden Einrichtung noch eine näher liegende rheumatologische Praxis vorhanden ist.

Bei den Luftliniendistanzen handelt es sich um einen approximativen Unterschied, welcher keinen Rückschluss auf die tatsächlich zurückgelegten Fahrtwege zulässt. Unter der Annahme eines proportionalen Verhältnisses zwischen Luftliniendistanz und Fahrweg lässt sich hierbei der regionale Versorgungsunterschied exakter darstellen. Für diese Auswertung konnten jedoch nur vertragsärztlich tätige FÄ für Rheumatologie berücksichtigt werden, nicht aber die in Klinikambulanzen tätigen FÄ. Diese übernehmen in einigen Regionen einen großen Versorgungsanteil, der hier nicht abgebildet ist. In den Daten der KV wird lediglich die Existenz mindestens einer rheumatologischen Praxis, nicht aber in der Anzahl oder anhand von Teil- und Vollzeitäquivalenten unterschieden. Gerade in bevölkerungsreichen Regionen mit großer FÄ-Dichte lassen sich Versorgungsdefizite nicht adäquat abbilden. Berücksichtigt werden sollte auch, dass die Postleitzahlregionen eine unterschiedliche Größe aufweisen, da sie neben der Einwohnerzahl auch auf historisch gewachsenen Faktoren wie Post-Zustellrouten und Ortschaften basieren. Aspekte der lokalen Infrastruktur (Zugang zu öffentlichen Verkehrsmitteln) und die Einwohnerzahl (mit Auswirkung auf Terminverfügbarkeiten) bleiben in dieser Auswertung unberücksichtigt. Die Auswertung ist also in die Richtung zu deuten, dass große Luftliniendistanzen einen Rückschluss auf Versorgungsdefizite nahelegen während kleine Luftliniendistanzen dennoch mit einem Versorgungsdefizit aus anderen Gründen einhergehen können.

## Fazit für die Praxis

In einigen Regionen Deutschlands müssen Patient:innen deutlich längere Anfahrtswege auf sich nehmen, um eine fachärztliche rheumatologische Versorgung zu erreichen. Es gibt aber auch viele Regionen, in der die Verteilung der FÄ für Rheumatologie ausgeglichen erscheint. Für die Betreuung in spezialisierten Zentren nehmen Patient:innen häufig weite Anfahrtswege in Kauf.
